# Validating survey measurement scales for AIDS-related knowledge and stigma among construction workers in South Africa

**DOI:** 10.1186/s12889-016-2756-z

**Published:** 2016-01-23

**Authors:** Paul Bowen, Rajen Govender, Peter Edwards

**Affiliations:** 1 Department of Construction Economics and Management, University of Cape Town, Private Bag, Rondebosch, 7701 Cape Town, South Africa; 2 Medical Research Council of South Africa and Department of Sociology, University of Cape Town, Private Bag, Rondebosch, 7701 Cape Town, South Africa; 3 School of Property, Construction and Project Management, RMIT University, Swanston Street, Melbourne, Victoria Australia

**Keywords:** HIV/AIDS, AIDS-related knowledge, AIDS-related stigma, Measurement scales, Construction workers, South Africa

## Abstract

**Background:**

Construction workers in South Africa are regarded as a high-risk group in the context of HIV/AIDS. HIV testing is pivotal to controlling HIV transmission and providing palliative care and AIDS-related knowledge and stigma are key issues in addressing the likelihood of testing behaviour. In exploring these issues, various studies have employed an 11-item AIDS-related knowledge scale (Kalichman and Simbayi, AIDS Care 16:572-580, 2004) and a 9-item stigma scale (Kalichman et al., AIDS Behav 9:135-143, 2005), but little evidence exists confirming the psychometric properties of these scales.

**Methods:**

Using survey data from 512 construction workers in the Western Cape, South Africa, this research examines the validity and reliability of the two scales through exploratory and confirmatory factor analysis and internal consistency tests.

**Results:**

From confirmatory factor analysis, a revised 10-item knowledge scale was developed (*χ*2 /*df* ratio = 1.675, CFI = 0.982, RMSEA = 0.038, and Hoelter (95 %) =393). A revised 8-item stigma scale was also developed (*χ*2 /*df* ratio = 1.929, CFI = 0.974, RMSEA = 0.045, and Hoelter (95 %) = 380). Both revised scales demonstrated good model fit and all factor loadings were significant (*p* < 0.01). Reliability analysis demonstrated excellent to good internal consistency, with alpha values of 0.80 and 0.74, respectively. Both revised scales also demonstrated satisfactory convergent and divergent validity. Limitations of the original survey from which the data was obtained include the failure to properly account for respondent selection of language for completion of the survey, use of ethnicity as a proxy for identifying the native language of participants, the limited geographical area from which the survey data was collected, and the limitations associated with the convenience sample. A limitation of the validation study was the lack of available data for a more robust examination of reliability beyond internal consistency, such as test-retest reliability.

**Conclusions:**

The revised knowledge and stigma scales offered here hold considerable promise as measures of AIDS-related knowledge and stigma among South African construction workers.

## Background

The South African construction industry employed approximately 490 000 people as at December 2014 [1], of which 190 000 were employed in the Western Cape [2]. The most recent South African National HIV Prevalence, Incidence and Behaviour Survey reports that South Africa has an overall national HIV prevalence of 12.2 % [3]. The construction industry has been identified as one of the sectors most adversely affected by HIV/AIDS [4–6]. In a nation-wide study of 10243 construction workers, it was reported that 13.9 % of that sample was HIV+ [7]. The high proportion of HIV+ persons in the construction sector is attributed to, *inter alia*, the fragmented nature of the construction industry [8]; its predominance of small firms; a significantly migratory workforce [9]; and the diversity of construction work in terms of the nature of the work itself, the types of projects, and the locations of construction sites [10]. Specifically, small firms generally do not have the resources to provide meaningful HIV prevention and treatment programmes. Rather, they tend to focus at best on awareness campaigns i.e., posters and dispensing condoms [11, 12]. In addition, the diversity of construction work makes it difficult to standardise or implement meaningful HIV interventions on construction sites. This is because the workforce changes frequently due to the nature of the production process, the number of sites to be covered, and the use of sub-contract and temporary labour. The construction industry is also one of the least responsive to the pandemic [11, 13]. Construction workers can thus be regarded as a high-risk group.

HIV testing is pivotal to controlling disease transmission and providing care [14–16]. A factor positively related to testing behavior in South Africa is a person’s level of AIDS-related knowledge [3, 17, 18]. Individuals with higher levels of correct knowledge about HIV/AIDS are far more likely to volunteer to be tested. Conversely, fear of AIDS-related stigma is a major barrier to HIV/AIDS testing, effective prevention and compliance with treatment regimes [19–22].

Several measures have been developed to explore AIDS-related stigma in southern Africa [23–28]. The development and testing of these measurement instruments is comprehensively summarised elsewhere [21]. Of the extant instruments, the scale developed by Kalichman et al. (2005) is particularly useful in that it was developed specifically for use in the general South African population, it has been previously validated, and is available in three of the country’s official languages [23].

In response to a perceived need for a validated and psychometrically sound multi-item measure for assessing AIDS-related stigma in South Africa, Kalichman et al. (2005) developed a 9-item scale [23]. Earlier work by Kalichman and Simbayi (2004), in the context of examining traditional beliefs about the cause of AIDS and AIDS-related stigma, used an 11-item AIDS-related *knowledge* scale in South Africa [29]. Several studies of the general population have since adopted this scale and the 2005 stigma scale as the bases for their measures of AIDS-related knowledge and stigma, respectively [30–32]. However, while these measures have been partially validated in the general population, their reliability and validity amongst construction workers is not known.

The aim of this study was to assess the psychometric properties of the HIV/AIDS knowledge and AIDS-related stigma scales developed by Kalichman and Simbayi (2004) and Kalichman et al. (2005) for assessing HIV/AID knowledge and AIDS-related stigma specifically in a sample of construction workers in South Africa [23, 29]. The research examined the validity of the scales through exploratory and confirmatory factor analysis, and their reliability by using tests of internal consistency. Additional validity tests for convergent and divergent validity are also performed.

## Background to the study

In a study into traditional beliefs as explanations for the cause of AIDS and AIDS-related stigma, Kalichman and Simbayi (2004) describe the development and application of an 11-item HIV/AIDS knowledge scale [29] adapted from the 18-item measure (HIV-KQ-18) reported by Carey and Schroder (2002) [33]. HIV-KQ-18 is itself a shorter version of a 45-item scale (HIV-K-Q) developed earlier [34]. All three measures reflect information about HIV casual contagion, HIV transmission/prevention, and HIV disease processes. For the 2004 Kalichman and Simbayi scale, adapted specifically for use in South Africa, item responses were coded as ‘Yes’, ‘No’, or ‘Don’t know’ [29]. This version of the AIDS knowledge test was scored for the number of correct answers, with ‘Don’t know’ being scored as incorrect. Higher scores indicated higher levels of HIV/AIDS-related knowledge. The surveys were self-administered in English and *isiXhosa* (an indigenous African language) to 487 men and women residing in a township in Cape Town, South Africa. No formal validation of this scale is available. The items contained in this scale are shown in Table 1.

**Table 1:** AIDS-related knowledge and stigma scale items and associated statistics (*n* = 457); depicts the AIDS-related knowledge and stigma scale items, correct/agree response statistics, scale item means, and standard deviations for the final dataset (*n* = 457)

Item	*n*	%	Mean	SD
	Correct/Agree			
AIDS knowledge scale (range 0–11)^a^				
1. Is AIDS spread by kissing? *(No)*	306	67	0.67	0.47
2. Can a person get AIDS by sharing kitchens or bathrooms with someone who has AIDS? *(No)*	329	72	0.72	0.45
3. Can you get AIDS by touching someone who has AIDS? *(No)*	356	78	0.78	0.42
4. Can men give AIDS to women? *(Yes)*	366	80	0.80	0.40
5. Can women give AIDS to men? *(Yes)*	389	85	0.85	0.36
6. Must a person have many different partners to get AIDS? *(No)*	228	50	0.50	0.50
7. Does washing after sex help protect against AIDS? *(No)*	318	70	0.70	0.46
8. Can a pregnant woman give AIDS to her baby? *(Yes)*	301	66	0.66	0.47
9. Can a person get rid of AIDS by having sex with a virgin? *(No)*	370	81	0.81	0.39
10. Is HIV the virus that causes AIDS? *(Yes)*	365	80	0.80	0.40
11. Is there a cure for AIDS? *(No)*	296	65	0.65	0.48
Mean	7.93			
SD	2.85			
AIDS-related stigma scale (range 0–9)				
1. People who have AIDS are dirty	49	11	0.11	0.31
2. People who have AIDS are cursed	37	8	0.08	0.27
3. People who have AIDS should be ashamed	69	15	0.15	0.36
4. It is safe for people who have AIDS to work with others, including children (*R*)	145	32	0.32	0.47
5. People who have AIDS must expect some restrictions on their freedom	196	43	0.43	0.50
6. A person with AIDS must have done something wrong and deserves to be punished	68	15	0.15	0.36
7. People who have HIV should be isolated	56	12	0.12	0.33
8. I do not want to be friends with someone who has AIDS	67	15	0.15	0.35
9. People who have AIDS should not be allowed to work	84	18	0.18	0.39
Mean	1.69			
SD	1.80			

*Notes*: ^a^Correct responses indicated in italics in parentheses against each knowledge question. (*R*) indicates item is reverse coded

Kalichman et al. (2005) also developed a scale to measure AIDS-related stigma in South Africa [23]. An initial pool of items was adapted from measures described by Pequegnat et al. (2001), Bauman et al. (2002), and Herek et al. (2002) [35–37], together with three scale items drawn from a National Institute of Mental Health (NIMH) International Collaborative HIV/STD Prevention Trial conducted in China, India, Peru, Russia and Zimbabwe. This initial pool comprised 24 items. The pool of 24 items was subsequently refined to a 9-item scale, with dichotomous response options as either ‘Agree’ or ‘Disagree’. Higher scores indicated higher levels of AIDS-related stigmatizing attitudes. No details were provided as to how the 9 items were selected, nor were explanations of the refinement process given. Items for this scale are shown in Table 1.

## Methods

A survey questionnaire, containing, *inter alia,* both sets of questions, was used to study a sample of on-site construction workers in the Western Cape province of South Africa. The sample was one of convenience. Construction firms that had participated in earlier HIV/AIDS research were approached and a subset of these agreed to participate in the current phase [11, 12]. These participating firms then identified current project construction sites in the greater Cape Town area for inclusion in the study. The number of sites and number of employees per site varied across participating firms.

### Ethical considerations

Prior, written ethical clearance was obtained from the University of Cape Town.

### Participants

Survey participants were 512 site-based employees from 6 firms on 18 sites in the Western Cape, South Africa. Most participants were male (91 %), and 62 % were permanent employees, as distinct from contract (employed on a project basis) (34 %) and occasional (casually hired) workers (4 %). Employment category was included given the relationship between category and HIV risk [7]. However, no information was available on employment (job) type. Participant age ranged from 18 to 69 years (mean = 36, SD = 10.86), with most respondents being in the 21–30 year age group. Almost two-thirds (62 %) of participants were ‘Black’ African (as distinct from ‘White’ or other ethnic groupings). Over a quarter (29 %) had at most primary level education, 52 % had secondary level education, and 19 % had tertiary education or higher. The questionnaire was made available in the three languages most commonly spoken in the province, and 40 % of completed questionnaires were returned in English, 14 % in *Afrikaans*, and 46 % in *isiXhosa.*

Twenty-seven percent of the sample reported that they had never been tested for HIV/AIDS, while 7 % reported themselves as having tested positively for HIV.

### Procedure

Each site population consisted of all employees present on the day and at the time of the visit by the researcher team. Participating workers were first briefed by the field administrators on the nature of the study, and assured that their participation was entirely voluntary and that they could withdraw such participation at their will. They were further assured that all responses would remain entirely anonymous and confidential, and that such responses would not be identifiable or impact their status with their employer. No payment was offered to participants. Following this briefing, participants who provided informed consent then proceeded to complete the questionnaires. Three workers at one site declined to participate, without providing reasons. At least three research assistants were present for each site visit and between them were proficient in all three survey-languages. The time taken by participants to complete the questionnaire varied between 30 min and 1 h. The site visits and associated data collection commenced in June 2013 and was completed by August 2013.

### Missing values and data analysis

The initial dataset comprised 512 returned questionnaires. Variables of interest for the evaluation of the two scales comprised the items that constitute the knowledge and stigma scales (see Table 1). Additionally, following Kalichman and Simbayi (2003) [38] and Kalichman et al. (2005) [23], variables used for construct validity analysis were also retained, namely, the education level of participants (‘primary or less’; ‘secondary’; or ‘tertiary or higher’), a belief that an HIV+ person should hide their status from others (‘agree’; or ‘disagree’), respondents feeling that they would rather not know if they were HIV+ (‘agree’; or ‘disagree’), a belief that people would abandon them if they contracted AIDS (‘agree’; or ‘disagree’), whether or not they had been tested for HIV (‘tested’; or ‘not tested’), and their HIV serostatus (HIV-; or HIV+). These additional items are not depicted in Table 1.

Missing value analysis on the knowledge items indicated that the proportion of the sample with missing values on each of the 11 items was less that 2 %. Similarly, for the stigma items, the proportion of the sample with missing values ranged from 2.5 to 3.3 %. The low frequency of missing values for items from both scales meant that these could be addressed by deletion of applicable cases – those missing items on either one or both scales. This resulted in a 457-case dataset (hereafter termed the ‘final dataset’) with no missing values for any knowledge or stigma items. The distribution of demographic characteristics in the final dataset is depicted in Table 2 and is almost identical to that in the original dataset of 512 cases.

**Table 2:** Characteristics of participants in the random split sub-samples A (*n* = 224) and B (*n* = 233); depicts the demographic characteristics, and the AIDS- related knowledge and stigma scale statistics for sub-samples A and B

Characteristics	Sample		Sub-sample A		Sub-sample B	
	(*n* = 457)		(*n* = 224)		(*n* = 233)	
	*n*	%	*n*	%	*n*	%
*Demographic characteristics*						
Age in years^c^	*M* = 36.09	*SD* = 10.95	*M* = 37.09	*SD* = 11.11	*M* = 35.16	*SD* = 10.74
Gender^b^						
Male	414	91	197	89	217	93
Female	40	9	24	11	16	7
Race/ethnicity^b^						
‘Others’	185	41	91	41	94	41
‘Black’ African’	270	59	132	59	138	59
Level of education^a^						
Primary or less	121	26	65	29	56	24
Secondary	245	54	110	49	135	59
Tertiary or higher	91	20	49	22	42	18
Nature of employment^a,d^						
Permanent	271	61	146	68	125	55
Temporary/Contract	152	34	65	30	87	38
Casual	21	5	5	2	16	7
Language^a^						
English	197	43	92	41	105	45
Afrikaans	67	15	35	16	32	14
isiXhosa	193	42	97	43	96	41
*HIV/AIDS-related characteristics*						
Tested for HIV^b^	339	74	167	75	172	74
HIV + ^b^	31	10	14	9	17	10
*Behavioural characteristics*						
**AIDS-related knowledge** ^c^	***Mean***	***SD***	***Mean***	***SD***	***Mean***	***SD***
Knowledge score (Range 0–11)	7.93	2.85	7.96	2.96	7.90	2.76
**AIDS-related stigma** ^c^	***Mean***	***SD***	***Mean***	***SD***	***Mean***	***SD***
Stigma score (Range 0–9)	1.69	1.80	1.70	1.86	1.68	1.74

*Notes*: ^a^The Chi-square test for independence or the ^b^Fisher’s Exact Test was used for categorical variables, and the ^c^Independent Samples ‘t’ Test was used for continuous variables. ^d^No differences were found between sub-sample characteristics and means, except for nature of employment; with sub-sample A containing a higher proportion of permanent workers than did sub-sample B

To enable the separate exploratory and confirmatory analyses, the final dataset was randomly divided into two discrete sub-samples. This is the recommended protocol for psychometric validation in a single sample [39]. Random split of the full dataset was performed using SPSS. The first sub-sample (A) contained 224 cases, whilst the second (holdout) sub-sample (B) contained 233 cases. The characteristics of both sub-samples are shown in Table 2. Analysis confirmed that both sub-samples were equivalent in terms of demographics and other key variables, an exception being with respect to nature of employment. Specifically, sub-sample A contained a significantly higher proportion of permanent workers than did sub-sample B. Unless otherwise stated, these datasets were used for all subsequent analyses.

For both the knowledge and stigma survey item responses, exploratory factor analysis (EFA) was applied to sub-sample A, whilst holdout sub-sample B was used for confirmatory factor analysis (CFA). The sub-sample sizes were sufficient for EFA and CFA [40, 41]. Knowledge and stigma *scales* were created by summating the scores of their respective items - reversed where appropriate given the direction of question wording.

### Statistical analysis

Using IBM SPSS version 22.0 for Macintosh [42], a variety of statistical analyses were performed on sub-sample A.

Once the suitability of the data for factor analysis was confirmed, the dimensionality of both scales was explored by means of exploratory factor analysis (EFA), with maximum likelihood estimation (ML) and Oblimin rotation. Following Pallant [43], three decision rules guided the number of factors to be retained: Kaiser’s criterion (eigenvalues exceeding 1) [44]; an inspection of the scree plot [45]; and Horn’s [46] parallel analysis (PA). PA was conducted using software developed by Watkins [47].

Following the EFA, confirmatory factor analysis (CFA) using maximum likelihood estimation to evaluate model fit was conducted on the holdout sub-sample B using IBM AMOS Version 22.0 for Windows [48]. Four critical fit indices were applied to determine the degree of fit of the structural equation models as follows (indices reflecting good model fit indicated in parenthesis): *χ* ^2^/df ratio (less than 4); Bentler CFI (comparative fit index (0.95 and greater)); RMSEA (root mean square error of approximation (0.05 and less)); and Hoelter (critical N (CN) index) (200 and greater) [49]. RMSEA is described as the most informative statistic in determining model fit as it takes cognizance of the number of variables being estimated in the model [50]. Model improvements and parsimony were tested using the Chi-Square Difference Test [51]. Following the EFA, the finalised SEM models were tested on the full final dataset (*n* = 457).

Once model development had been concluded, the scales were subject to reliability testing using the test of internal consistency (Cronbach’s alpha). Scale scores were then developed to enable testing of convergent and divergent validity of the scales using the additional items described above.

## Results

### Descriptive statistics

For the final dataset (*n* = 457), participant scores on the summated knowledge scale ranged from 0 to 11, with a mean of 7.93 (SD = 2.85) and a median of 9.0. Higher scores indicated higher levels of HIV/AIDS-related knowledge. The summated stigma scale scores ranged from 0 to 9, with a mean of 1.69 (SD = 1.80) and median of 1.0. Higher scores indicated higher levels of AIDS-related stigmatizing attitudes. Details of knowledge and stigma scale item responses, means, and SDs are shown in Tables 1 and 2. The stigma score statistics obtained for the final dataset (Table 1: mean = 1.69 and SD 1.8) correspond almost exactly with those reported by Kalichman et al. (2005) from their sample of 2306 participants (mean score 1.7 and SD 1.9) [23].

### Bivariate correlation analysis

Using the sub-sample A (*n* = 224), Pearson’s bivariate correlation analysis was performed on the items contained in each of the two scales. Without exception, all knowledge items were significantly positively correlated, mostly at *p <* 0.01. Similarly, with the stigma items, the majority were significantly positively associated, many at *p* < 0.01; the sole exception being the item ‘*safe to work with others and children*’. This particular item did not correlate significantly with any other stigma item.

### Exploratory factor analysis

Exploratory factor analysis for both scales was undertaken on sub-sample A (*n* = 224). To assess the suitability of the sample for factor analysis, Bartlett’s Test of Sphericity (BToS) and the Kaiser-Meyer-Olkin (KMO) measure of sampling adequacy were used. For both the knowledge scale and stigma scale items, the Bartlett’s tests were significant (*χ* ^2^ = 795.35, *df* = 55, *p* < 0.01; and *χ* ^2^ = 395.96, *df* = 36, *p* < 0.01, respectively). The KMO values supported the suitability of the data for factor analysis (KMO = 0.844 for the knowledge scale and KMO = 0.829 for the stigma scale).

For the knowledge scale items, the EFA revealed two factors with eigenvalues exceeding 1, explaining 30.1 and 12.6 % of the variance, respectively. This coincided with only two factors exceeding the criterion values generated by PA (100 replications). Following Oblimin rotation, the factors displayed a moderate, negative inter-factor correlation (*r* = −0.50). Table 3 depicts the pattern and structure matrices. Inspection of the pattern matrix shows a relatively clear two-factor solution, with the exception of the ‘*virus*’ item. Importantly, this item loads almost equally onto the two factors (0.310 and −0.294, respectively).

**Table 3:** Factor analysis of AIDS-related knowledge and stigma scale items (*n* = 224); depicts the pattern and structure matrices for ML with oblimin rotation for the knowledge and stigma scale items

Item	Factor 1		Factor 2	
	Pattern	Structure	Pattern	Structure
AIDS knowledge scale (2-factor solution)				
Does washing after sex help protect against AIDS?	**.703**	**.696**	.014	-.340
Can a person get AIDS by sharing kitchens or bathrooms with someone who has AIDS?	**.666**	**.669**	-.007	-.343
Is AIDS spread by kissing?	**.630**	**.552**	.154	-.163
Can you get AIDS by touching someone who has AIDS?	**.591**	**.653**	-.123	-.421
Can a person get rid of AIDS by having sex with a virgin?	**.550**	**.614**	-.126	-.404
Is there a cure for AIDS?	**.540**	**.533**	.013	-.259
Can a pregnant woman give AIDS to her baby?	**.425**	**.534**	-.216	-.430
Must a person have many different partners to get AIDS?	**.357**	**.362**	-.010	-.190
Is HIV the virus that causes AIDS?	**.310**	**.458**	-.294	-.450
Can men give AIDS to women?	.009	.481	**-.936**	**-.941**
Can women give AIDS to men?	.025	.443	**-.830**	**-.842**
AIDS-related stigma scale (1-factor solution)				
A person with AIDS must have done something wrong and deserves to be punished	**.699**	**.688**	-.152	-.102
People who have HIV should be isolated	**.674**	**.685**	.151	.199
People who have AIDS are cursed	**.652**	**.614**	-.534	-.487
People who have AIDS are dirty	**.627**	**.613**	-.198	-.153
I do not want to be friends with someone who has AIDS	**.582**	**.604**	.303	.345
People who have AIDS should be ashamed	**.495**	**.499**	.045	.080
People who have AIDS should not be allowed to work	**.442**	**.446**	.059	.091
People who have AIDS must expect some restrictions on their freedom	**.341**	**.347**	.077	.102
It is safe for people who have AIDS to work with others, including children (*R*)	.042	.053	**.154**	**.157**

*Note*: (*R*) indicates item is reverse coded. For oblique rotations (oblimin in SPSS) the loadings and correlations are distinct. The pattern matrix depicts the loadings of the variables on the factors. Each row of the pattern matrix is essentially a regression equation where the standardized observed variable is expressed as a function of the factors. The loadings are the regression coefficients. The structure matrix holds the correlations between the variables and the factors

For the stigma scale items, the EFA revealed one factor with an eigenvalue exceeding 1, explaining 29.17 % of the variance, and this was confirmed by the criterion values generated by PA (100 replications). Table 3 depicts the pattern and structure matrices. All items except one clearly loaded onto the factor - the exception being the item dealing whether it is ‘*safe for HIV+ persons to work with others, including children*’. When this item was removed, the EFA returned a stable single factor solution accounting for 32.1 % of the variance. The contribution of this item to the scale was explored more fully in the CFA.

Overall, these results supported the underlying dimensionality of the two scales (with the exclusion of the ‘*safe to work with others, including children*’ item in the stigma measure). A concern emanating from this EFA was the cross-loadings of the ‘*virus*’ item onto both factor 1 and factor 2. These issues were explored more fully in the CFA.

### Confirmatory factor analysis

Confirmatory factor analysis using maximum likelihood estimation was conducted on the holdout sub-sample B (*n* = 233). A number of alternative models were investigated, for both the knowledge and stigma scale items.

For the knowledge scale, an 11-item two-factor model as identified in the EFA was investigated, allowing the factors to correlate freely. Model indices indicated a reasonably good fit to the data (*χ*2 /*df* ratio = 1.761, CFI = 0.943, RMSEA = 0.057, and Hoelter (95 %) = 182). Factor loadings in this model were all statistically significant (*p* < 0.01). Examination of the residuals and modification indices revealed no further modification to improve model fit.

Although the EFA indicated a two-factor model, given the previously identified dimensionality of the items, the suitability of one-factor model was explored. This model was a poor fit (*χ*2 /*df* ratio = 4.464, CFI = 0.736, RMSEA = 0.122, and Hoelter (95 %) = 72). However, all factor loadings in this model were statistically significant (*p* < 0.01). The factor loadings are standardized path coefficients interpreted in the same manner as the loadings in the pattern matrix of the EFA. Inspection of the modification indices indicated the need for correlating the errors for the items *‘sharing kitchens’* and *‘touching’*, as well as for the *‘men-to-women’* and *‘women-to-men’* transmission items. With these paths specified, the resultant model proved a very good fit (*χ*2 /*df* ratio = 1.519, CFI = 0.962, RMSEA = 0.047, and Hoelter (95 %) =212). Overall, this model was much more robust, improving significantly on the initial one factor model and marginally on the initial two factor model.

For the stigma data a 9-item one-factor model as identified in the EFA was investigated. The model displayed good fit to the data: *χ*2 /*df* ratio = 1.472, CFI = 0.954, RMSEA = 0.045, Hoelter (95 %) =235. The factor loadings of all items were statistically significant (*p* < 0.01), except for the ‘*safe to work with others, including children*’ item (*p* = 0.234). The modification indices recommended permitting correlation of the error terms for the ‘*dirty*’ and ‘*not friends*’ items. With this modification, the revised model improved *χ*2 /df ratio = 1.196, CFI = 0.981, RMSEA = 0.029, Hoelter (95 %) = 291. The Chi-Square Difference Test confirmed the statistically significant improvement in the revised model Δχ^2^(1) = 8.646, *p* < 0.01. Again, all factor loadings of all items were statistically significant (*p* < 0.01), except for the item ‘*safe to work with others, including children*’ (*p* = 0.249). Given the lack of statistical significance of the factor loading for the ‘*safe to work with others, including children*’ item, the model was re-run with this item omitted. The resultant model fit was a very good fit: *χ*2 /df ratio = 1.355, CFI = 0.976, RMSEA = 0.039, and Hoelter (95 %) = 272. The Chi-Square Difference Test revealed that this model was not a significant improvement on the previous model [(Δχ^2^(7) = 5.342, *p* > 0.05), indicating that the inclusion/exclusion of this item did not substantively enhance/diminish the model. Consequently, this item was excluded from the final stigma scale.

### Reliability analysis

Using the holdout sub-sample B (*n* = 233), the reliability of both scales as developed in the CFA was assessed by means of the Cronbach’s alpha internal consistency statistic. The analysis indicated very good internal consistency for the knowledge scale (α = 0.79) and good internal consistency for the stigma scale (α = 0.71), with no evidence to suggest the removal of any item from either scale.

### Confirmatory factor analysis using the full final dataset

To test the robustness of the revised AIDS knowledge and stigma scales, both SEM models developed using the holdout sub-sample B (*n* = 233) were tested using the full final dataset (*n* = 457).

For the knowledge scale, good model fit was achieved for a single factor solution: *χ*2 /*df* ratio = 2.247, CFI = 0.960, RMSEA = 0.052, and Hoelter (95 %) = 281, and all item-factor loadings were statistically significant (*p* < 0.01). However, the modification indices indicated the need for correlation amongst the error terms of the *‘kissing’* and the *‘sharing kitchens’* items. Once this path was specified, the model fit improved substantially: *χ*2 /*df* ratio = 1.936, CFI = 0.971, RMSEA = 0.045, and Hoelter (95 %) =328. The Chi-Square Difference Test confirmed the statistically significant improvement in the second model Δχ^2^(1) = 15.00, *p* < 0.01.

**Fig. 1:**
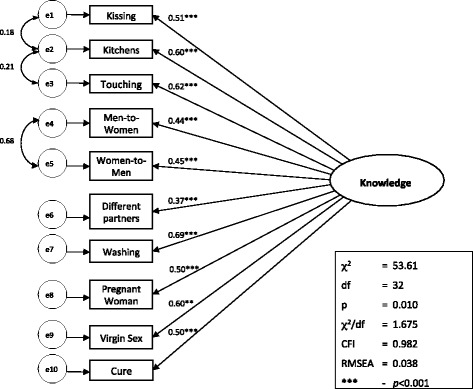
Confirmatory factor analysis of final AIDS-related knowledge scale; this figure depicts the structural equation model (SEM) for the final AIDS-related knowledge scale

Finally, given the lack of a strong loading by the ‘*virus*’ item onto a single factor as indicated in the EFA, the model was re-run with this item omitted. The resultant model fit was a very good fit: *χ*2 /*df* ratio = 1.675, CFI = 0.982, RMSEA = 0.038, and Hoelter (95 %) =393. Again, the Chi-Square Difference Test confirmed the better fit of this model against the model including the item (Δχ^2^(9) = 25.76, *p* < 0.01). Based on these results, and in the interests of parsimony, the *‘virus’* item was excluded from the final knowledge scale. The final 10-item, one-factor AIDS knowledge scale is depicted in Fig. 1.

**Fig. 2:**
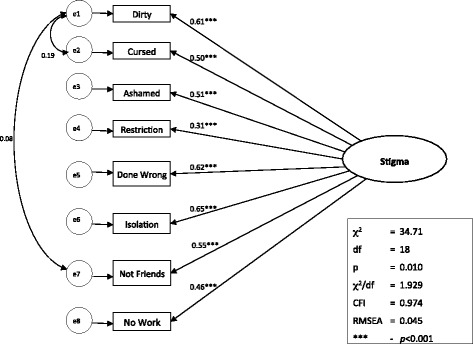
Confirmatory factor analysis of final AIDS-related stigma scale; this figure depicts the structural equation model (SEM) for the final AIDS-related stigma scale

For the revised stigma model, reasonably good model fit was achieved on the full final dataset: *χ*2 /*df* ratio = 2.363, CFI = 0.959, RMSEA = 0.055, and Hoelter (95 %) = 307. All item-factor loadings were statistically significant (*p* < 0.001). Examination of the modification indices indicated the need to allow the error terms for the *‘dirty’* and *‘cursed’* items to correlate. Upon such specification, the resultant model improved considerably: *χ*2 /*df* ratio = 1.929, CFI = 0.974, RMSEA = 0.045, and Hoelter (95 %) = 380. The Chi-Square Difference Test confirmed that the second model was a significant improvement over the first: Δχ^2^(1) = 10.18, *p* < 0.01. The final 8-item, one-factor AIDS stigma scale is depicted in Fig. 2.

### Reliability analysis of the final scales using the full final dataset

Using the final dataset (*n* = 457), the Cronbach’s alpha statistic was computed for the revised knowledge and stigma scales. The analysis returned an excellent reliability estimate for the revised knowledge scale (α = 0.80) and a good estimate for the revised stigma scale (α = 0.74).

#### Language effects for scale reliability

Language has been known to impact the reliability of measurement scales in two principal ways: 1) the specific language in which a scale is provided to respondents, and 2), the discrepancy between the native language of the respondent and the language in which they completed a questionnaire.

Examination of the reliability estimates for the two scales as a function of the language in which the survey questionnaires were completed revealed some variation in scale reliability estimates. For the AIDS knowledge scale, the estimates of internal consistency were: α = 0.67 (English), α = 0.74 (*Afrikaans*), and α = 0.79 (*isiXhosa*). There are no previous internal consistency estimates by language for this scale to serve as a basis for comparison. The AIDS-related stigma scale differentiation by language returned the following estimates: α = 0.67 (English), α = 0.66 (*Afrikaans*), and α = 0.74 (*isiXhosa*). These broadly align with those of Kalichman et al. (2005) [23].

The language of testing has particular relevance if it differs from the native language of the respondent in that any such variation is likely to diminish the reliability of the scale. The construction worker survey from which this data was obtained had not clearly established the home language of participants, so the validation study used ethnicity as a proxy for this. Cross-tabulation of ethnicity against the language of the completed questionnaires revealed that 74 (27 %) ‘Black’ African participants (who in all likelihood have *isiXhosa* as their native language) had opted to fill in an English language questionnaire. This group accounted for 38 % of participants who completed an English questionnaire. The remaining participants who completed the English version were not ‘Black” African. For ‘Black’ African participants, a significant relationship exists between level of education and the language of the questionnaire. Specifically, greater levels of education are associated with increased use of an English questionnaire. Better-educated ‘Black’ African workers are therefore more likely to be fluent in English, and may thus have preferred to complete an English questionnaire.

To examine for the effect of this native-test language disjuncture on the reliability of the scales, additional analyses were performed on both revised scales. Firstly, reliability analysis was performed using participants who had completed an English questionnaire and who had stipulated their ethnicity as ‘Black’ African. For these respondents the alpha value for the revised knowledge scale was α = 0.58. In contrast, the alpha value for the remaining ethnic groups was α = 0.72. The difference in the reliability of the scale across the two groups of respondents is quite pronounced, and indicates that the reliability estimate obtained for the entire sample was effectively lowered by the ‘Black’ African participants who chose to complete their questionnaires in English rather than their native language. This is further confirmed by the fact that the reliability estimate for the knowledge scale for ‘Black’ African respondents who had completed the questionnaire in their native language is higher than either of these two reliability estimates: α = 0.79.

A similar reliability analysis was performed in respect of the revised stigma scale, and indicated marginal differences. For the questionnaires completed in English, the overall alpha value was α = 0.67. For the English questionnaires completed by ‘Black’ African participants, the alpha value for the revised stigma scale was α = 0.69, compared to an α = 0.66 for the remaining participants who completed an English questionnaire. The difference between the estimates, while small, seems to suggest that the scale works better for ‘Black’ African participants - regardless of the language in which they completed the scale – than it does for non-‘Black’ African respondents. This is supported by the fact that the reliability estimate for this scale for ‘Black’ African respondents who had completed this scale in their native language is higher: α = 0.72. A possible explanation for this may be the cultural specificity of the scale, as discussed later on.

Taken together, the language based reliability analyses indicates that the overall reliability for both scales as established earlier on effectively masks some degree of variation as a result of either the variation in the test language, the discrepancy between this test language and the native language of the respondent, or both. This effect is much more pronounced for the knowledge scale than for the stigma scale.

### Convergent and divergent validity of the final scales using the full final dataset

For the convergent and divergent validity analysis of the revised AIDS knowledge and stigma scales, the following items were utilised: educational level, ‘hide HIV status’, ‘rather not know’, ‘abandon me’, HIV status (positive or negative) and HIV testing (tested or not). These items were variously drawn from Kalichman and Simbayi (2003) [38] and Kalichman et al. (2005) [23].

The correlation between the revised AIDS knowledge and stigma scales, and between each of these scales with the education level of participants was examined in the final dataset (*n* = 457). Knowledge and stigma were found to be significantly inversely correlated (*r* = −0.28, *n* = 457, *p* < 0.01), as were education level and stigma (*r* = −0.37, *n* = 457, *p* < 0.01). Individuals with lower levels of education were more likely to exhibit higher levels of stigma towards AIDS and HIV+ persons. Education was found to be significantly positively correlated with knowledge about AIDS (*r* = 0.40, *n* = 457, *p* < 0.01), indicating that higher levels of education were associated with more informed positions with respect to HIV and AIDS. These results broadly align with those of Kalichman et al. (2005) [23].

Participants who agreed with the statement that infected persons should *‘hide their status’* scored significantly lower on the AIDS knowledge scale [agree (*M* = 6.40, SD = 2.66) and disagree (*M* = 7.27, SD = 2.63); *t*(455) = 2.63, *p* < 0.01], and significantly higher on the stigma scale [agree (*M* = 2.45, SD = 2.42) and disagree (*M* = 1.16, SD = 1.46); *t*(85) = −4.49, *p* < 0.01].

Persons who agreed with the *‘rather not know’* statement scored significantly lower on the AIDS knowledge scale [agree (*M* = 6.27, SD = 2.60) and disagree (*M* = 7.31, SD = 2.63); *t*(453) = 3.08, *p* < 0.01], and significantly higher on the stigma scale [agree (*M* = 2.62, SD = 2.43) and disagree (*M* = 1.13, SD = 1.44); *t*(82) = −5.06, *p* < 0.01].

Participants who agreed with the *‘abandon me’* statement scored significantly lower on the AIDS knowledge scale [agree (*M* = 6.26, SD = 2.87) and disagree (*M* = 7.43, SD = 2.52); *t*(165) = 3.84, *p* < 0.01] and significantly higher on the stigma scale [agree (*M* = 2.33, SD = 2.30) and disagree (*M* = 1.05, SD = 1.34); *t*(132) = −5.50, *p* < 0.01]. This finding aligns with Kalichman et al. (2005) in respect of the stigma scale, but no similar evaluation by them was done for the knowledge scale [23].

HIV+ workers scored significantly lower on the AIDS knowledge scale [HIV+ (*M* = 6.26, SD = 3.03) and HIV- (*M* = 7.44, SD = 2.48); t(34) = −2.10, *p* < 0.05], but no association was found between HIV status and AIDS-related stigma scores.

Workers who had previously tested for HIV/AIDS scored significantly higher on the AIDS knowledge scale [Tested (M = 7.29, SD = 2.57) and Not Tested (*M* = 6.69, SD = 2.85); t(454) = −2.11, *p* < 0.05], but again no association was found between HIV testing and AIDS-related stigma scores.

Overall, both the stigma and the knowledge scales were able to differentiate individuals who were significantly different from one another in terms of their education, views on HIV+ persons hiding their status, preferring to not know if they were HIV+, feeling that they would be abandoned if they contracted AIDS, whether or not they had been tested for HIV/AIDS, and their HIV serostatus.

## Discussion

As examined herein, the optimal HIV/AIDS knowledge scale is a 10-item scale, while the optimal HIV/AIDS stigma scale is an 8-item scale. Table 4 defines the items in each of the final scales. In terms of validity, both scales were found to be strongly unidimensional, while in terms of reliability, both scales were found to be internally consistent to a good to very good degree.

**Table 4:** English language versions of items in the final AIDS-related knowledge and stigma scales

Item	
AIDS knowledge scale (Yes/No/Don’t know)	
1. Is AIDS spread by kissing?	
2. Can a person get AIDS by sharing kitchens or bathrooms with someone who has AIDS?	
3. Can you get AIDS by touching someone who has AIDS?	
4. Can men give AIDS to women?	
5. Can women give AIDS to men?	
6. Must a person have many different partners to get AIDS?	
7. Does washing after sex help protect against AIDS?	
8. Can a pregnant woman give AIDS to her baby?	
9. Can a person get rid of AIDS by having sex with a virgin?	
10. Is there a cure for AIDS?	
AIDS-related stigma scale (Agree/Disagree)	
1. People who have AIDS are dirty	
2. People who have AIDS are cursed	
3. People who have AIDS should be ashamed	
4. People who have AIDS must expect some restrictions on their freedom	
5. A person with AIDS must have done something wrong and deserves to be punished	
6. People who have HIV should be isolated	
7. I do not want to be friends with someone who has AIDS	
8. People who have AIDS should not be allowed to work	

Additionally, both scales were found to be significantly correlated with factors (level of education) with which they are assumed to be logically related (convergent validity) and each of them also successfully differentiated between groups of individuals who differed significantly in terms of their agreement on whether or not those with HIV should hide their status, agreement on whether or not they would rather not know if they were HIV positive, whether or not they felt they would be abandoned if they were to be infected with AIDS (divergent validity), whether or not they had been tested for HIV/AIDS, and their HIV serostatus. Where applicable, given the existence of previous research, these results correspond favourably with earlier findings [23].

Overall, the results confirm the underlying reliability and validity of the two revised scales. However, this validation study has some limitations. Internal consistency was used as the sole measure of scale reliability. Practical limitations precluded the use of other forms of reliability - particularly test-retest reliability - that would have arguably provided a more robust test of the scales. The convergent and divergent validity tests, while strongly supportive of the scales, would undoubtedly have benefitted from the use of criteria such as behaviourial measures rather than only attitudinal or demographic variables.

Additionally, the original study from which the data was obtained has some limitations. Firstly, the survey instrument failed to account for the choice exercised by respondents in terms of the specific language version of the questionnaire that they completed. As was clearly demonstrated, discrepancy between the test language and the native language of the ‘Black’ African respondents adversely affected the reliability of the revised AIDS knowledge scale for the entire sample. For the revised stigma scale, the results were less clear, suggesting a slightly higher reliability for ‘Black’ African respondents who completed the survey in their native language as compared to respondents from other ethnic groups. The overall direction suggests that the stigma scale might be far more reliable in communities that favour traditional views and beliefs about AIDS and HIV as compared to communities where less traditional and more scientific explanations are favoured. However, as the magnitude of the difference observed for the stigma scale was marginal, it is not advisable to read too much into this finding.

The native language-test language variation issue should nevertheless be flagged for special attention in further research, particularly that conducted in multilingual settings. In this regard four issues are critical: 1) the actual choice exercised by respondents in terms of the test language for the completion of the questionnaire, 2) attention to translation methods for multilingual versions of the scales, to ensure greater conceptual correspondence between translated versions, and 3) a more direct examination of how the overall sample reliability estimates may be masking significant differentiation in such estimates in language-defined sub-samples. Additionally, it is recommended that the language analysis also be extended to validity testing, as it is likely that the findings of variable scale reliability estimates are derivative of variable scale validity as a function of language.

Furthermore, the data from the original study were obtained from construction workers on sites in the greater Cape Town metropolitan area, and are not therefore representative of the population of on-site construction workers in other provinces, or the country as a whole. Given the variability in ethnicity, language and cultural beliefs and values across different regions of the country, it remains for future research to determine whether or not the dimensionality and reliability of the scales as established here will prove invariant in different samples, not only for construction workers but also for the communities from which these workers emanate.

Finally, the limitations associated with convenience sampling need to be acknowledged. Participating workers employed by co-operating construction firms who have previously participated in HIV/AIDS research are likely to be more knowledgeable about HIV/AIDS, and are less likely to be prejudicial towards and discriminatory against HIV+ persons.

## Conclusions

In the context of HIV/AIDS, construction workers in South Africa are regarded as a high-risk group. HIV testing is pivotal to controlling HIV transmission and providing palliative care and AIDS-related knowledge and stigma are key issues in addressing the likelihood of testing behaviour. This study examined the psychometric properties of the 11-item knowledge and 9-item stigma scales developed by Kalichman and Simbayi (2004) [29] and Kalichman et al. (2005) [23] as applied to a sample of construction workers in South Africa. The statistical examination of both measures returned strong evidence confirming the validity and reliability of each of the scales. From confirmatory factor analysis, a revised 10-item knowledge scale and a revised 8-item stigma scale were developed. Both revised scales also demonstrated satisfactory convergent and divergent validity. Limitations of the original survey from which the data was obtained include the failure to properly account for respondent selection of language for completion of the survey, use of ethnicity as a proxy for identifying the native language of participants, the limited geographical area from which the survey data was collected, and the problems associated with the convenience sample. A limitation of the validation study was the lack of available data for a more robust examination of reliability beyond internal consistency, such as test-retest reliability. Despite these limitations, the revised knowledge and stigma scales offered here hold considerable promise as measures of AIDS-related knowledge and stigma among South African construction workers.
